# p90RSK Regulates p53 Pathway by MDM2 Phosphorylation in Thyroid Tumors

**DOI:** 10.3390/cancers15010121

**Published:** 2022-12-25

**Authors:** Immacolata Maietta, Francesca Del Peschio, Preziosa Buonocore, Eleonora Viscusi, Stefano Laudati, Giuseppe Iannaci, Michele Minopoli, Maria Letizia Motti, Valentina De Falco

**Affiliations:** 1 Institute of Endocrinology and Experimental Oncology (IEOS), National Research Council (CNR), Via S. Pansini 5, 80131 Napoli, Italy; 2 Department of Molecular Medicine and Medical Biotechnology (DMMBM), University of Naples “Federico II”, Via S. Pansini 5, 80131 Naples, Italy; 3 Complex Operative Unit, Pathological Anatomy, Pellegrini Hospital ASL NA1, 80134 Naples, Italy; 4 Complex Operative Unit, Pathological Anatomy “Ospedale del Mare” ASL NA1 80147 Naples, Italy; 5 Preclinical Models of Tumor Progression, Department of Translational Research Supporting Oncological Pathways-National Cancer Institute of Naples, IRCCS “G. Pascale” Via M. Semmola, 80131 Naples, Italy; 6 Department of Movement Sciences and Wellbeing, University “Parthenope”, Via Medina 40, 80133 Naples, Italy

**Keywords:** p90RSK, MDM2, proteasome degradation, p53, cancer, cell proliferation, apoptosis, tumorigenesis, targeted therapy

## Abstract

**Simple Summary:**

p90RSK is a downstream effector protein of the MAPK pathway. In many human cancers, the MAPK pathway is constitutively activated due to oncogenic mutations of its components. Consequently, p90RSK is hyperactive and capable of hyper phosphorylating substrates involved in tumorigenesis. p90RSK belongs to the AGC kinase family, which phosphorylates substrates with an RXRXXS/T consensus motif. MDM2 protein presents this consensus in its sequence at serine 166. MDM2 is a ubiquitin ligase that negatively regulates the protein stability of p53. The aim of this study has been to verify whether p90RSK is capable of phosphorylating MDM2 in serine 166 and to investigate the role of p90RSK in regulating the p53 pathway, especially in thyroid tumors in which the MAPK pathway is constitutively active and in which the development of specific drugs aimed at inhibiting the kinase activity of p90RSK could represent a new way of inhibiting tumorigenesis.

**Abstract:**

The expression level of the tumor suppressor p53 is controlled by the E3 ubiquitin ligase MDM2 with a regulatory feedback loop, which allows p53 to upregulate its inhibitor MDM2. In this manuscript we demonstrated that p90RSK binds and phosphorylates MDM2 on serine 166 both in vitro and in vivo by kinase assay, immunoblot, and co-immunoprecipitation assay; this phosphorylation increases the stability of MDM2 which in turn binds p53, ubiquitinating it and promoting its degradation by proteasome. A pharmacological inhibitor of p90RSK, BI-D1870, decreases MDM2 phosphorylation, and restores p53 function, which in turn transcriptionally increases the expression of cell cycle inhibitor p21 and of pro-apoptotic protein Bax and downregulates the anti-apoptotic protein Bcl-2, causing a block of cell proliferation, measured by a BrdU assay and growth curve, and promoting apoptosis, measured by a TUNEL assay. Finally, an immunohistochemistry evaluation of primary thyroid tumors, in which p90RSK is very active, confirms MDM2 stabilization mediated by p90RSK phosphorylation.

## 1. Introduction

**Figure 1:**
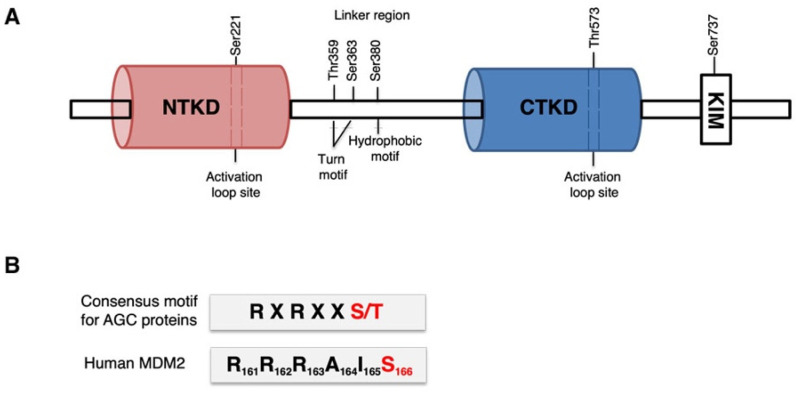
(**A**) Schematic representation of p90RSK structure. The N-terminal kinase domain (NTKD), the C-terminal kinase domain (CTKD), and the linker region were highlighted. The main phosphorylation sites were indicated, and the numbers refer to the RSK1 isoform; (**B**) representation of the consensus motif for AGC kinase family [7]. The S/T phosphorylation site is highlighted in red. The consensus sequence individuated in human MDM2 by www.phosphosite.org site (accessed on 14 November 2014) was indicated with the amino acid’s numbers.

The 90 kDa ribosomal S6 kinase (p90RSK) is a family of highly conserved serine–threonine kinases including four human isoforms, RSK1, 2, 3, and 4. RSKs are characterized by two kinase domains: the N-terminal kinase domain (NTKD), belonging to an AGC family of kinases (for similarity of catalytic domain found in PKA, PKG, and PKC enzymes), which is the effector domain of the kinase and responsible for the phosphorylation of substrates containing a basophilic consensus motif (Arg/Lys-X-Arg-X-X-Ser/Thr or Arg-Arg-X-Ser/Thr) [1,2] and the C-terminal kinase domain (CTKD), which is a calcium/calmodulin-dependent protein kinase with a regulatory function; they are separated by a linker region. The C-terminal tail contains a regulatory docking site for ERK (KIM: kinase-interacting motif) (Figure 1A) [3,4]. Activation of RSKs is mediated by a coordinated response to different stimuli: growth factors, neurotransmitters, hormones and mitogen-activated protein kinases (MAPKs) [5,6]; they activate phosphorylation on different residues sequentially, including Thr573, Thr359, Ser363, Ser380, and Ser221 (the numbers refer to the RSK1 isoform) (Figure 1A) [7]. The currently accepted model of p90RSK activation maintains that ERK1/2 kinases phosphorylate p90RSK on threonine 573, within the CTKD, and on T359 and S363, within the linker region. Phosphorylation of T573 leads to the activation of CTKD, which in turn phosphorylates S380 that binds PDK1. PDK1 consequently phosphorylates S221 in NTKD, resulting in its activation. Furthermore, following its dissociation from PDK1, the S380 can bind to a hydrophobic site of NTKD, stabilizing it and achieving its complete activation. Finally, the autophosphorylation of Ser737 determines the dissociation of ERK and the possibility for activated p90RSK to bind and phosphorylate its substrates [1,7,8].

p90RSK regulates the MAPK pathway with negative feedback, which starts with p90RSK and limits the activation of the RAS/ERK signaling cascade. Inhibition of p90RSK with BI-D1870 increases ERK1/2 phosphorylation in different cell types, suggesting that the physiological function of p90RSK is to prevent overactivation of the MAPK pathway [8].

Being able to control the transcription of many proteins, in particular of various transcription factors, among them CREB, ERalpha, and IkB [9,10,11], p90RSK regulates multiple cellular processes: protein synthesis, gene expression, cell cycle and growth, survival, proliferation, migration, and differentiation [12,13,14]. 

Interestingly, p90RSK is involved in tumorigenesis and the first works on this topic are already ten years old. The four p90RSK isoforms showed different involvement in various tumor types. RSK1 and RSK2 isoforms were found to be strongly expressed in prostate and breast carcinomas [15] and osteosarcoma [16]; moreover, RSK2 promotes metastases of head and neck squamous cell carcinoma (HNSCC) [17]. 

RSK3 and RSK4 isoforms mediate resistance to PI3K inhibitors in breast cancer [18]; furthermore, p90RSK4 has been shown to be involved in sunitinib resistance in renal cell carcinoma and melanoma cell lines [19]. However, to date, drugs targeted to block p90RSK are not yet used in cancer clinics, despite the great variety of functions regulated by the different p90RSK isoforms and the fact that the chemical inhibition of this group of kinases has been shown to be effective in blocking the invasion and metastasis of several solid tumors in preclinical models [20], and even though there are many more recent examples of p90RSK involvement in tumor pathogenesis and resistance to conventional therapies. 

Ubiquitination is a post-translational modification in which the proteins to be degraded are labeled with ubiquitin and then degraded through the proteasome. Ubiquitin is bound to lysine residues on proteins by three different enzymes: E1 activates ubiquitin, E2 combines ubiquitin, and E3 is the ubiquitin ligase that transfers ubiquitin to target proteins [21,22]. Mouse double minute 2 (MDM2) is an E3 ubiquitin ligase that ubiquitinates p53, inducing its translocation into the cytoplasm and its proteasome-dependent degradation, making it unable to activate target gene transcription [23,24]. Furthermore, MDM2 is capable of self-ubiquitinating and promoting its own degradation [25]. 

p53 itself is responsible for the transcription of MDM2, which once translated and activated interacts with p53, causing its ubiquitination. This mechanism generates an autoregulatory feedback loop that tightly regulates the function of p53: p53 positively regulates MDM2 expression and MDM2 negatively regulates p53 [26]. p53 controls cell cycle arrested by the transcriptional modulation of the cyclin-dependent kinase inhibitor p21Waf1/Cip1 [27]. Furthermore, several apoptotic genes are transcriptionally regulated by p53, among them Bax, a pro-apoptotic member of the BCL-2 family [28] and Bcl-2 [29]. It is already known that MDM2 activity is regulated by AKT by serine (S) 166 phosphorylation. The S166 is present in the RXRXXS/T AKT consensus motif and its phosphorylation induces MDM2 stabilization [30]. 

We have identified the MDM2/p53 pathway as a novel target of p90RSK since this kinase phosphorylates MDM2 on S166, stabilizing it and allowing it to ubiquitinate p53, promoting its degradation. We also showed that p90RSK, via MDM2/p53, downregulates Bax and p21 and upregulates Bcl-2 expression, influencing cell proliferation and apoptosis. Since p90RSK is strongly activated in many human tumors [7], the use of specific drugs targeted to inhibit its kinase activity could represent a very important mechanism because the inhibition of p90RSK is able to stimulate, through the regulation of MDM2 and p53, the block of the proliferation and the promotion of apoptosis in cancer cells.

## 2. Materials and Methods

### 2.1. Compounds

BI-D1870 (Selleckchem, Houston, TX, USA) was dissolved in DMSO at a concentration of 10 mM and stored at −80 °C. 

MG132 (Calbiochem, San Diego, CA, USA) was dissolved in DMSO at 10 µM and stored at −20 °C. 

Cycloheximide (SIGMA, Darmstadt, Germany) was dissolved in DMSO at a concentration of 25 mg/mL and stored at 4 °C. 

Nutlin3a (SIGMA, Darmstadt, Germany) was dissolved in DMSO at 10 mM and stored at −20 °C. 

### 2.2. Cell Lines and Transfection

HEK293 and HeLa cells were from the American Type Culture Collection (ATCC, Rockville, MD, USA). Transient transfections were carried out with the FuGENE HD reagent according to manufacturer’s instructions (Roche, Mannheim, Germany).

Six human thyroid cell lines were used in the study:

Human papillary thyroid cancer TPC1 cells were obtained in 1990 from M. Nagao (National Cancer Center Research Institute, Tokyo, Japan) and expresses RET/PTC1 rearrangement [31,32]. The TPC1 used was identified based on the unique presence of the RET/PTC1 rearrangement, the most common genetic alteration identified to date in thyroid papillary carcinomas.

Human thyroid carcinoma 8505C and CAL62 cells were purchased from DSMZ (Deutsche Sammlung von Mikroorganismen und Zellkulturen GmbH, Braunschweig, Germany). Human thyroid carcinoma SW1736 cell was obtained by N.E. Heldin (University Hospital, S-751 85 Uppsala, Sweden). Human thyroid carcinoma BCPAP cells were obtained from the primary source (N. Fabien, CNRS URA 1454, University of Medecine Lyon-Sud, Oullins, France). 8505C cells, established from an anaplastic thyroid carcinoma containing areas of papillary thyroid carcinoma, harbor homozygous BRAF V600E mutations and NF2 (p.Glu129Ter), TERT (c.1-146C > T), and TP53 (p.Arg248Gly) heterozygous mutations.

CAL62 cells, established from an anaplastic thyroid carcinoma, harbor CREBBP (p.Glu1541Ter), EP300 (p.Asp1485fs), KRAS (p.Gly12Arg), NF2 (p.Glu215Ter), and TP53 (p.Ala161Asp) mutations.

SW1736 cells, established from an anaplastic thyroid carcinoma, harbor homozygous TP53 (p Gln192Ter) mutations and BRAF (pVal600Glu), TERT (c.1-124C > T), and TSHR (p. Ile486Phe) heterozygous mutations.

BCPAP cells, established from poorly differentiated thyroid gland carcinoma (PDTC), harbor homozygous BRAF (p Val600Glu) and TP53 (p.Asp259Tyr) mutations and TERT (c.1-124C > T) heterozygous mutation.

The information on the mutations was retrieved from “https://web.expasy.org/cellosaurus” (Accession date: 23 May 2022).

8505C, SW1736, and CAL62 were DNA profiled by a short tandem repeat (STR) analysis and shown to be unique and identical to those reported in Schweppe et al., 2008 [33].

Nthy-ori 3-1 (thyroid follicular epithelial cells, further referred to as NTHY), a cell line derived from normal human thyroid follicular cells transformed by SV40 virus [33], was obtained from the European Collection of Authenticated Cell Cultures, ECACC.

All the cell lines were grown in DMEM (Dulbecco’s Modified Eagle Medium) with 10% fetal calf serum (Invitrogen, Waltham, MA, USA). Medium was supplemented with 2 mM L-glutamine and 100 units/mL penicillin-streptomycin (Invitrogen). The cell lines were regularly tested for the absence of mycoplasma infections.

Transient transfections of TPC1 were carried out with the Lipofectamine 2000 reagent according to manufacturer’s instructions (Invitrogen).

### 2.3. Cell Proliferation Assay

For cell proliferation assays, about 100 × 10^3^ TPC1 and 200 × 10^3^ SW1736 cells were plated in 60 mm dishes. The day after plating, the cells were counted and BI-D1870 or vehicle were added. The cells were counted in triplicate at the indicated time points and the number of cells at 72 h of treatment was used as a reference to calculate the IC50.

### 2.4. Plasmids 

Plasmids encoding p90RSK1 were cloned in the pRK7-myr expression vector (Addgene, Teddington, UK). Plasmids encoding p90RSK2 were cloned in the pWZL-neo-myr-flag expression vector (Addgene, Teddington, UK). Plasmids encoding p53 were cloned in the pcDNA3 expression vector (Addgene, Teddington, UK). Plasmids encoding MDM2 and MDM2 S166A mutant (pcDNA3 MDM2wt and pcDNA3 MDM2S166A) were a kind gift from Professor Robin Fahraeus (INSERM, Paris, France). The plasmid encoding MDM2 S166D mutant (pcDNA3 MDM2 S166D) was a kind gift from Dr. Fabiola Moretti (CNR, Rome, Italy).

### 2.5. Protein Extraction, Western Blotting, and Antibodies

Cells were harvested in JS lysis buffer (50 mM Hepes, pH 7.5, 150 mM NaCl, 10% glycerol, 1% Triton X-100, 1 mM EGTA, 1.5 mM MgCl_2_, 10 mM NaF, 10 mM sodium pyrophosphate, 1 mM Na_3_VO_4_, 10 µg of aprotinin/mL, 10 µg of leupeptin/mL) and clarified by centrifugation at 10,000× *g*. Protein concentration was calculated with a modified Bradford assay (Bio-Rad Laboratories, Berkeley, CA, USA). Antigens were revealed by an enhanced chemiluminescence detection kit (ECL, Amersham Pharmacia Biotech, Amersham, UK). Signal intensity was quantified using ImageJ software 1.53q. RSK1 and MDM2 immunoprecipitation was performed respectively with an anti-RSK1 (sc-231) and anti-MDM2 (SMP-14, sc-965) antibody, according to datasheets instructions. Total lysates and immunoprecipitates were separated by SDS–PAGE and then transferred on to nitrocellulose filters (Schleicher & Schuell). Membranes were blocked with 5% BSA, 1× TBS, 0.1% Tween-20 and probed with the different antibodies. Anti-phospho-S166-MDM2 (#3521), anti-phospho-S102-YB1 (#2900), anti-RSK1/2/3 (#6195), anti-Bax (#2772), anti-Bcl-2 (#2870), and anti-phospho-T308-AKT (#9275) were from Cell Signaling Technologies (Danvers, MA, USA). Anti-RSK1 (sc-231), anti-RSK2 (sc-9986), anti-p53 (sc-126), anti-MDM2 (SMP-14, sc-965), anti-p21 (sc-397) were from SantaCruz Biotechnology (Dallas, TX, USA). Monoclonal anti-α-tubulin (#T9026) was from Sigma Aldrich (St Louis, MO, USA). Secondary antibodies coupled to horseradish peroxidase were from Amersham Pharmacia Biotech. Each sample in the immunoblots was quantized with the ImageJ program and expressed as a ratio to the amount of the protein used to normalize (supplementary Figures).

### 2.6. In Vitro Kinase Assay 

RSK active (SignalChem, Richmond, Canada. cat. R15-10G, 10 µg/100 µL) at final concentration of 200 ng in 20 µL, ATP at final concentration of 50 µM in 20 µL and MDM2 (Recombinant Human GST MDM2/HDM2, cat. E3-202, BostonBiochem, Boston, MA, USA, 10 µM) at final concentration of 20 ng in 20 µL were used. 

The experiment took place in the appropriate Kinase Assay Buffer (25 mM MOPS pH 7.2, 12.5 mM beta-glycerophosphate, 25 mM MgCl_2_, 5 mM EGTA, 2 mM EDTA, 1 M DTT). The samples were incubated with the kinase 15′ at 30 °C, then they were incubated 15′ with ATP, centrifuged 5′ at 2000 rpm, washed with kinase assay buffer, and finally prepared for Western blot. The experiment was repeated using BI-D1870 at a concentration of 10 μM, 5 μM, and 1 μM by adding BI-D1870 after adding the ATP for 15′. The phosphorylated substrates were separated on SDS–PAGE and signal intensity was analyzed using ImageJ software 1.53q.

### 2.7. RNA Extraction and Real-Time PCR 

Total RNA from the indicated samples was prepared using the RNeasy Midi Kit (Qiagen, Crawley, WestSussex, UK) and subjected to on-column DNase digestion with the RNase-free DNase set (Qiagen, Crawley, West Sussex, UK) following the manufacturer’s instructions. The quality of RNA from each sample was verified by electrophoresis through 1% agarose gel and visualized with ethidium bromide.

cDNA was synthesized in a 50 μL reaction volume starting from 2.5 µg RNA by using the Gene Amp RNA PCR Core Kit (Applied Biosystems, Warrington, UK). Quantitative polymerase chain reactions (qPCR) were performed by using the SYBR Green PCR Master mix (AppliedBiosystems, Waltham, MA, USA) in the iCycler apparatus (Bio-Rad, Munich, Germany). Amplification reactions (25 mL final reaction volume) contained 200 nM of each primer, 3 mM MgCl2, 300 µM dNTPs, 1 × SYBR Green PCR buffer, 0.1 U/µL AmpliTaq Gold DNA polymerase, 0.01 U/µL Amp Erase, RNase-free water, and 2 µL cDNA samples. Thermal cycling conditions were optimized for each primer pair (80 cycles starting from 55 °C for 10 s with increments of 0.5 °C). Primers were designed by using a software available at https://www.ncbi.nlm.nih.gov/tools/primer-blast/ (accessed on 13 October 2022). Primer sequences were as follows:

p53 Forward: TTCTTGCATTCTGGGACAGCC;
Reverse: GCTTCTGACGCACACCTATTG;
forward: AAGATGCGCGGGAAGTAGC;
Reverse: GGTTTTGGTCTAACCTGGAGGC;
GAPDH forward: CCATCACCATCTTCCAGGAGC;
Reverse: AGAGATGATGACCCTTTTGGC.

### 2.8. TUNEL Assay 

For the TUNEL, an equal number (100 × 10^3^) of TPC1 was seeded onto single well with Costar L-polylisine-treated glass slides. After 72 h treatment with 4 µM BI-D1870 or vehicle, cells were fixed in 4% (*w*/*v*) paraformaldehyde and were then permeabilized by the addition of 0.1% Triton X-100/0.1% sodium citrate. Slides were rinsed twice with PBS and subjected to the TUNEL reaction (Boehringer, Mannheim, Germany). All coverslips were counterstained in PBS containing Hoechst 33258 (final concentration, 1 µg/mL; Sigma ChemicalCo., St. Louis, MO, USA), rinsed in PBS and mounted in PBS 1X-Glycerol on glass slides. The fluorescent signal was visualized with an epifluorescent microscope (Axiovert2, Carl Zeiss Vision GmbH, Munchen, Germany) (equipped with a 10× objective) interfaced with the image analyzer software KS300 (Zeiss) version 4.6. At least 100 cells were counted in five different microscopic fields. 

### 2.9. BrdU Assay and Immunofluorescence

We used a bromodeoxyuridine (BrdU) labeling and detection kit from Boehringer Mannheim (Germany). Cells were seeded on glass slides, incubated for 1 h with BrdUrd (final concentration of 10 µmol/L), fixed, and permeabilized with ethanol-glycine solution. Slides were incubated with anti-BrdU mouse monoclonal antibody and with a fluorescein (FITC)-conjugated secondary antibody (Boehringer Mannheim, Mannheim, Germany) and mounted in PBS 1X-Glycerol on specimen holder slide.

For immunofluorescence, MDM2 was revealed with an anti-MDM2 primary antibody and fluorescein (FITC)-conjugated secondary antibodies (Jackson Immuno Research Laboratories, Inc., Philadelphia, PA, USA). Cell nuclei were identified by Hoechst 33258 (final concentration of 1 µg/mL; Sigma ChemicalCo.) staining. The fluorescent signal was visualized with an epifluorescent microscope (Axiovert 2, Zeiss; equipped with a ×100 lens) interfaced with the image analyzer software KS300 (Zeiss). At least 100 GFP-positive cells were counted in five different microscopic fields. 

### 2.10. RNA Silencing

The small inhibitor duplex RNAs (siRNA) were from Dharmacon and were ON-target plus SMARTpool siRSK1 Human: #L-003025-00 and siRSK2 Human: #L-003026-00. The siCONTROL Non-Targeting Pool (#D-001206-13-05) was used as a negative control. Cells were transfected with 100 nmol/L siRNAs using DharmaFECT reagent. The day before transfection, cells were plated in 35 mm dishes at 40% of confluence in DMEM supplemented with 10% FBS without antibiotics. 

### 2.11. Histological Examination and Immunohistochemistry

Thyroid samples were from “Ospedale del Mare” (ASL NA 1 Centro, Naples, Italy). Thyroid carcinomas were classified according to the America Joint Committee on Cancer (AJCC) TNM system. All patients agreed to make their tumor tissue available for research studies. This study was approved by the Internal Reviewing Board. Normal and tumor thyroid specimens were fixed in 10% neutral buffered formalin, embedded in paraffin, and 4 μm sections were stained with hematoxylin–eosin according to standard procedures.

MDM2 expression and RSK phosphorylation were detected by incubating the slides, respectively, with the mouse monoclonal antibody anti-MDM2 (clone IF2) #337100 (Invitrogen) and the mouse monoclonal antibody phospho-RSK2 sc-374664 (SantaCruz Biotechnology).

Immunoreactions were displayed by the avidin–biotin–peroxidase complex (ABC) method (Ultraview Universal DAB Detect kit Ventana, Roche, Basel, Switzerland) by a Benchmark Immunostainer (Ventana, Tucson, AZ, USA). Negative controls were carried out by omitting the primary antibodies.

The sections were observed under a light microscope and photographed using a digital scanner (Ventana DP 200, Roche) at 40× magnification.

### 2.12. Statistical Analysis 

For the statistical analysis, the one-way ANOVA with standard parametric methods and the Bonferroni Multiple Comparison Test (InStat program, GraphPad software 3.1) and χ2 analysis with Fisher’s exact test were performed. Data were analyzed with the standard statistical software SPSS 20 (SPSS Inc., IBM, New York, NY, USA). *p* values were statistically significant at *p* < 0.05.

## 3. Results

### 3.1. p90RSK Directly Binds and Phosphorylates MDM2 on S166 In Vitro

As previously mentioned, the N-terminal domain (NTKD) of p90RSK belongs to a family of AGC kinases and is responsible for the phosphorylation of substrates containing the consensus motif (RXRXXS/T) (Figure 1B) [7]. Analysis of the human MDM2 protein sequence revealed a serine residue, S166, within amino acids 161–166 (RRRAIS), which corresponds to the minimum consensus for p90RSK-mediated phosphorylation (Figure 1B). 

**Figure 2:**
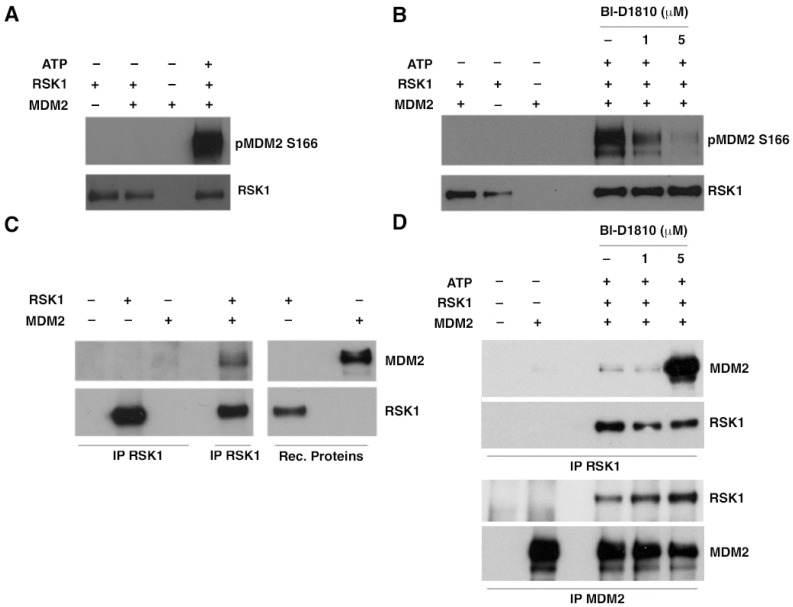
p90RSK binds and phosphorylates MDM2 on serine 166 in vitro. (**A**) An in vitro kinase assay was performed by using recombinant constitutively active RSK1 and recombinant MDM2. The immunoblot was analyzed with anti-phospho-S166 MDM2 antibody. To normalize for the amount of RSK1 the different samples, blots were probed with anti-RSK1 antibody; (**B**) in vitro kinase assay with or without different doses of BI-D1870. BI-D1870 inhibitor was added to the samples 10 min before ATP addition to the reaction mix; (**C**) in vitro immunoprecipitation experiment with recombinant RSK1 and MDM2 proteins. Recombinant proteins were immunoprecipitated with anti-RSK1 antibody and the immunocomplex was revealed with anti-MDM2; (**D**) in vitro immunoprecipitation experiment with recombinant RSK1 and MDM2 proteins in the presence or not of different doses of BI-D1870. Recombinant proteins were immunoprecipitated with anti-RSK1 antibody or anti-MDM2 antibody and the immunocomplex was revealed with anti-MDM2 and anti-RSK1.

To determine whether p90RSK actually phosphorylates MDM2 on serine 166, we performed a kinase assay with the active recombinant p90RSK1 and recombinant MDM2 protein. Using an anti-p-S166 antibody, we demonstrated that p90RSK phosphorylates serine 166 of MDM2 (Figure 2A). To validate the specificity of the anti-p-S166 antibody signal we performed a kinase test using increasing concentration (1 and 5 µM) of a p90RSK reversible inhibitor, BI-D1870; the phosphorylation of S166 was strongly reduced and its reduction was correlated with the increase in inhibitor concentration and therefore with the p90RSK activity inhibition (Figure 2B). Since many kinases interact with their substrates, we investigated whether it also occurred between MDM2 and p90RSK1, immunoprecipitating recombinant proteins with anti-RSK1 and immunoblotting with anti-MDM2. The co-immunoprecipitation of the recombinant proteins, RSK1 and MDM2, was confirmed (Figure 2C). Furthermore, co-immunoprecipitation assay with anti-RSK1 antibody or anti-MDM2 antibody of recombinant proteins, using increasing concentration of BI-D1870 (1 and 5 µM) showed that the dephosphorylation of MDM2 increases its interaction with RSK1 (Figure 2D).

These results demonstrated that p90RSK directly interacts and phosphorylates MDM2 on S166 in vitro, in fact the use of the p90RSK inhibitor BI-D1870 causes a decrease in the phosphorylation of S166. Furthermore, this phosphorylation induces a decrease in the interaction between MDM2 and RSK1.

### 3.2. p90RSK Binds and Phosphorylates MDM2 on S166 In Vivo

**Figure 3:**
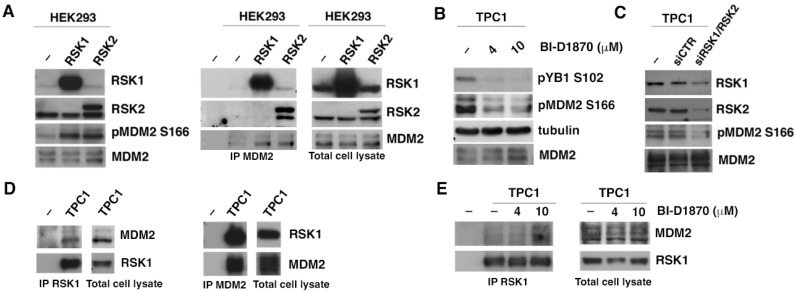
p90RSK binds and phosphorylates MDM2 on S166 in vivo. (**A**) Left panel—HEK293 cells were transfected with RSK1 and RSK2 expression plasmids. The transfection efficiency was verified by immunoblot with anti-RSK1 and anti-RSK2. The MDM2 phosphorylation level was assessed with the anti-phospho-S166 of MDM2. The experiment was normalized for total MDM2 (**A**) right panel—HEK293 cells were transfected with the plasmids encoding constitutively activated p90RSK1 and p90RSK2. The transfection efficiency was verified with anti-RSK1 and anti-RSK2 immunoblot. The experiment was normalized for total MDM2 (right panel). Proteins were immunoprecipitated with anti-MDM2 antibody and immunoblotted with anti-RSK1 and anti-RSK2 antibodies to verify the interaction and with anti-MDM2 antibody to normalize the immunoprecipitation (left panel); (**B**) TPC1 cells were treated with the indicated doses of BI-D1870 inhibitor. BI-D1870 inhibition efficiency was tested with the anti-phospho-S102 YB1 antibody. Phosphorylated MDM2 was revealed with anti-phospho-S166 MDM2 antibody. MDM2 total level was revealed with anti-MDM2 antibody. Normalization was performed by immunoblotting with anti-tubulin antibody; (**C**) TPC1 cells were transfected with RNA interference against RSK1 and RSK2 (pool of specific RSK1/RSK2 siRNAs) or with control RNA interference (siCTR). The interference efficiency was verified with anti-RSK1 and anti-RSK2 antibodies. Phosphorylated MDM2 was revealed with anti-phospho-S166 MDM2 antibody. Normalization was performed by immunoblotting with anti-MDM2 antibody; (**D**) co-immunoprecipitation experiments of the endogenous MDM2 and RSK1 proteins in TPC1 cells. Anti-RSK1 antibody (left panel) and anti-MDM2 antibody (right panel) were used as baits; the first lane represents the control IgG. Anti-RSK1 antibody (left panel) and anti-MDM2 antibody (right panel) were used to verify the efficiency of immunoprecipitation. Anti-MDM2 antibody (left panel) and anti-RSK1 antibody (right panel) were used to verify the interaction between RSK1 and MDM2; (**E**) co-immunoprecipitation experiment of RSK1 and MDM2 in TPC-1 cells treated for 1 h with the indicated doses of BI-D1870. Anti-RSK1 antibody was used for immunoprecipitation (left panel). The first lane represents the control IgG. Anti-RSK1 antibody was used to verify the efficiency of immunoprecipitation and anti-MDM2 antibody to verify changes in the interaction between RSK1 and MDM2 (left panel). Normalization of total proteins was performed by anti-RSK1 and anti-MDM2 antibodies (right panel).

By transient transfection of HEK293 cell line with plasmids expressing constitutively active myristilated RSK1 and RSK2 [34], we demonstrated that endogenous MDM2 is phosphorylated by both kinases on S166 (Figure 3A, supplementary Figure S3A, left panel) and that both kinases bind MDM2 in co-immunoprecipitation assay with anti-MDM2 antibody (Figure 3A, supplementary Figure S3A, right panel). Myristylated p90RSK is constitutively active because it binds to the plasma membrane by myristylation, which allows for complete activation of the kinase, bypassing the initial steps of ERK-dependent translocation. [34]. Using TPC1 cell line system, a papillary thyroid carcinoma cell line in which p90RSK is activated by oncogenic activation of upstream MAPK pathway due to RET/PTC1 rearrangement [35], we verified that endogenous p90RSK is able to phosphorylate MDM2 on S166 and that pharmacological inhibition of p90RSK with increasing concentrations of BI-D1870 (4, 10 μM) determines a progressive dephosphorylation of MDM2 on S166 (Figure 3B, supplementary Figure S3B). As a control of the efficacy of p90RSK inhibition, phosphorylation on S102 of YB-1, a known substrate of p90RSK [7], was assessed (Figure 3B, supplementary Figure S3B). Furthermore, to confirm that S166 of MDM2 was specifically phosphorylated by p90RSK, we performed a Western blot in TPC1 cells, after 72 h of silencing of RSK1 and RSK2 with a pool of specific siRNAs. The interference against RSK1 and RSK2 determines a decrease in S166 phosphorylation of MDM2 (Figure 3C, supplementary Figure S3C). To test whether endogenous p90RSK was able to interact directly with MDM2, as we previously demonstrated for recombinant proteins and transfected kinases, we immunoprecipitated total proteins from TPC1 cells with anti-RSK1 or with anti-MDM2 antibodies and in both cases, we highlighted the presence in the complex of MDM2 and RSK1, respectively (Figure 3D, supplementary Figure S3D). Moreover, we showed that the binding affinity between MDM2 and RSK1 is inversely correlated to the kinase activity: increment in concentration of BI-D1870 used to inhibit p90RSK (4, 10 μM) increases the amount of MDM2 bound to p90RSK showing that, once phosphorylated, MDM2 dissociates from the kinase (Figure 3E, supplementary Figure S3E). The experiments carried out on TPC1 were performed for other thyroid tumor lines (CAL62, BCPAP, SW1736, 8505C) and for a normal thyroid cell line (NTHY), and in all tumor cell lines the results are in agreement with those obtained in TPC1 (supplementary Figures S1 and S2). In normal thyroid cell lines, in which p90RSK is less active, the amount of MDM2 is reduced compared to tumor thyroid cells (supplementary Figure S1B). 

In conclusion, p90RSK binds and phosphorylates MDM2 onto S166 in vivo, resulting in the release of phosphorylated MDM2 from the RSK/MDM2 complex.

### 3.3. p90RSK Promotes Degradation of p53 via MDM2 Phosphorylation

**Figure 4:**
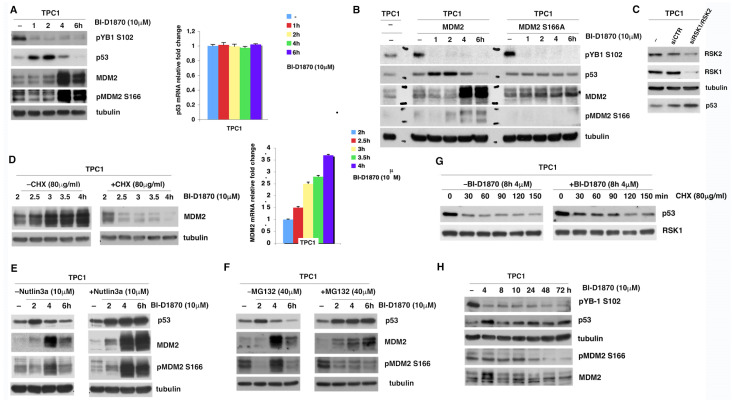
p90RSK promotes degradation of p53 via MDM2 phosphorylation. (**A**) TPC1 cells were treated with 10 μM BI-D1870 at the indicated times. The efficacy of the treatment was verified with anti-phospho-S102 YB1 antibody. The immunoblot was then tested with anti-p53, anti-MDM2 and anti-phospho-S166 antibodies and normalization was performed by immunoblotting with anti-tubulin antibody (left panel); analysis of p53 mRNA levels by quantitative RT-PCR in TPC1 cells treated with BI-D1870 at the indicated times. The experiment was conducted in triplicate and the standard deviations are represented by the diagram error bars (right panel); (**B**) TPC1 cells were transfected with MDM2wt or MDM2 S166A expression vectors, after 24 h cells were starved for 12 h and then treated with 10 μM BI-D1870 for the indicated times (h). The efficiency of transfection was verified with anti-MDM2 antibody compared with non-transfected TPC1 cells (−). The effectiveness of the treatment was verified with anti-phospho-S102 YB1 antibody. The immunoblot was then tested with anti-p53 and anti-phospho-S166 MDM2 antibodies and normalization was performed by immunoblotting with anti-tubulin antibody; (**C**) TPC1 cells were transfected with RNA interference against RSK1 and RSK2 (pool of specific RSK1/RSK2 siRNAs) or with control RNA interference (siCTR). The interference efficiency was verified with anti-RSK1 and anti-RSK2 antibodies. p53 total level was revealed with anti-p53 antibody. Normalization was performed by immunoblotting with anti-tubulin antibody; (**D**) TPC1 cells were pretreated with 10 µM BI-D1870 for 2 h and co-treated, or not, with cycloheximide at indicated times. The immunoblot was tested with anti-MDM2 antibody and normalized with anti-tubulin antibody (left panel); analysis of MDM2 mRNA levels by quantitative RT-PCR in TPC1 cells treated with BI-D1870 at the indicated times. The experiment was conducted in triplicate and the standard deviations are represented by the diagram error bars (right panel); (**E**) TPC1 cells were treated with BI-D1870 10 μM with or without Nutlin-3 at the indicated times. The immunoblot was tested with anti-p53, anti-MDM2, and anti-phospho-S166 antibodies and normalized with anti-tubulin antibody; (**F**) TPC1 cells were treated with 10 μM BI-D1870 with or without MG132 at the indicated times. The immunoblot was tested with anti-p53, anti-MDM2, and anti-phospho-S166 antibodies and normalized for tubulin; (**G**) TPC1 cells were treated with BI-D1870 4 μM for the total hours indicated (8 h) and treated with cycloheximide (CHX) for different times (min). The immunoblot was tested with anti-p53 antibody and normalized with anti-RSK1 antibody; (**H**) TPC1 cells were treated with 4 µM BI-D1870 for the indicated times (h). The efficacy of the treatment was verified with anti-phospho-S102 YB1 antibody. The immunoblot was then tested with anti-p53, anti-MDM2, and anti-phospho-S166 MDM2 antibodies and normalized for tubulin.

In order to explore whether p90RSK-induced MDM2 phosphorylation on serine 166 was important for the stability of p53, TPC1 cells were treated with 10 μM BI-D1870 for different periods of time (1, 2, 4, 6 h); in these cells, the turn-over of p53, being in wild-type form, is controlled by MDM2. After 1 and 2 h of treatment with 10 μM BI-D1870 the phosphorylation of S166-MDM2 gradually decreases, resulting in a progressive increase in p53. However, after 4 and 6 h of treatment with BI-D1870 the increase in p53 determines a notable upregulation of MDM2, presumably consisting of newly synthesized MDM2. Consequently, the phosphorylation level of serine 166 increases and, in turn, MDM2 is able to induce p53 degradation. The efficiency of p90RSK inhibition by BI-D1870 was verified by monitoring YB-1 serine 102 phosphorylation (Figure 4A, left panel). 

To investigate whether the regulation of p53 levels directly depended on the S166 phosphorylation of MDM2 by p90RSK, we transfected HeLa—cells with high transfection efficiency in which p53 activity is not constitutive and partially modulated by MDM2 due to the presence of HPV E6 [36]—with expression vectors for MDM2 wild-type (HeLa/MDM2wt), MDM2 S166A (HeLa/MDM2 S166A), a S166 phosphorylation-deficient mutant [37], or MDM2 S166D (HeLa/MDM2 S166D), a S166 phospho-mimetic mutant [37], and treated the transfected cells with BI-D1870 for different times (1, 2, 4, 6 h) (supplementary Figure S6). In HeLa/MDM2wt and HeLa/MDM2 S166D, BI-D1870 treatment induces an accumulation of p53 (supplementary Figure S6, left and right panel). This doesn’t occur in HeLa/MDM2 S166A (supplementary Figure S6, central panel), demonstrating that the regulation of p53 by p90RSK appears to be affected by MDM2 phosphorylation on S166. 

To confirm the data obtained on HeLa cells in human thyroid cancer cells, we transfected TPC1 with the MDM2 phosphorylation-deficient mutant (MDM2 S166A) or with the wild-type MDM2 (MDM2) (Figure 4B). The result shows that the regulation of p53 levels directly depends on the p90RSK-mediated phosphorylation of MDM2 S166; indeed, the treatment with BI-D1870 does not affect p53 levels in the presence of the mutant, demonstrating that the S166 phosphorylation is essential for p90RSK-dependent regulation of p53 (Figure 4B).

To establish the relative contribution of PI3K/AKT activation, compared to MAPK/p90RSK, on the MDM2/p53 pathway we analyzed the influence of AKT activation on S166 phosphorylation and, consequently, on p53 stability, by treating TPC1 cells with Wortmannin and LY-294002 for different times. Intriguingly, the results showed that in TPC1 cells the inhibition of p90RSK compared to that of AKT, although causing a similar inhibition of MDM2 phosphorylation on S166, affect most strongly the stabilization of p53 protein (supplementary Figure S7).

To confirm that the increase in p53 levels, after BI-D1870 treatment, was dependent on protein accumulation due to the blockade of MDM2-dependent degradation and was independent of transcriptional upregulation, quantitative analysis of p53 mRNA was performed by real-time PCR at different treatment times with BI-D1870 10 µM and compared with p53 protein levels indicated in Figure 4A, left panel. The results show that changes in p53 levels are independent by change in amount of its mRNA (Figure 4A, right panel). To demonstrate that the increment in p53 is dependent on the p90RSK-mediated reduction of MDM2 phosphorylation and independent of a possible nonspecific stress effect of BI-D1870 on TPC1 cells, we performed a genetic silencing of p90RSK by transfecting these cells with a pool of specific RSK1/RSK2 siRNAs or with control siRNA (siCTR). An increase in p53 levels occurs 10 h after silencing of p90RSK, confirming the hypothesis that the increase in p53 is determined by the inactivation of p90RSK (Figure 4C). 

Furthermore, to demonstrate that the increase in MDM2 after 2 and 4 h of treatment with BI-D1870 was dependent on the transcriptional activity of p53, TPC1 cells were maintained in the presence or absence of the protein synthesis inhibitor cycloheximide (CHX) and treated with BI-D1870 for different periods of time included in the 2/4 h range (2, 2.5, 3, 3.5, 4 h) (Figure 4D, left panel). The results show that the presence of cycloheximide blocks the accumulation of MDM2 instead of causing a decrease; inhibition of protein synthesis prevents translation of MDM2 mRNA, confirming that MDM2 accumulation was dependent on the transcriptional regulation of p53. The decrease in MDM2 is also justified by its higher turnover due to the low S166 phosphorylation caused by BI-D1870 treatment [38]. To reinforce these data and demonstrate that MDM2 changes are transcription-dependent, we measured MDM2 mRNA levels by RT-PCR in TPC1 cells treated with BI-D1870 in the same time frame in which an increase in MDM2 levels occurs. It is confirmed that treatment with BI-D1870 between 2 and 4 h induces an activation of the transcription of MDM2 (Figure 4D, right panel). By inhibition of the MDM2 activity with Nutlin-3a, an inhibitor of the interaction between MDM2 and p53, after different periods of treatment with 10 µM BI-D1870 (2, 4, 6 h), we have shown that in TPC1 cells treated with Nutlin3a the degradation of p53, expected after 4 and 6 h of treatment with BI-D1870, was inhibited, showing that it depends on p53/MDM2 interaction (Figure 4E). Moreover, use of MG132, an inhibitor of proteasome activity, determines a stabilization of p53 at 4 and 6 h of treatment with BI-D1870, demonstrating that MDM2-dependent p53 degradation is caused by the activity of the proteasome (Figure 4F); moreover, importantly, BI-D1870 decreases MDM2 due to its degradation by the proteasome as the use of MG132 causes its increase; consequently, this demonstrates that the phosphorylation of MDM2 by p90RSK on serine 166 stabilizes MDM2.

These experiments confirm the existence of a regulatory feedback loop, which allows p53 to transcriptionally regulate its MDM2 inhibitor and, in turn, the p90RSK-dependent serine 166 phosphorylation of MDM2, stabilizing it, promotes p53 degradation (Figure 4A).

Thus, to demonstrate that p90RSK was able to induce the degradation of p53 by MDM2 phosphorylation, the TPC1 cells, pre-treated for 8 h with 4 µM BI-D1870, were treated with cycloheximide for different period of time (30, 60, 90, 120, 150 min). The results show that the inhibition of p90RSK increases the amount of the p53 protein that is usually strongly reduced in TPC1 cells due to a high turnover mediated by constitutive activation of p90RSK (Figure 4G). p53 quantification, normalized for RSK1, is represented in the supplementary materials (supplementary Figure S4G, lower panel). Moreover, by treating the TPC1 cells with BI-D1870 for 1 h, in the presence of CHX for times from 0 to 60 min (supplementary Figure S4G, right panel), we have thoroughly shown that the half-life of p53 is extended by BI-D1870.

In conclusion, the block of protein synthesis by cycloheximide shows that the increase in p53 is due to its stabilization and is not dependent on the biosynthesis of new protein. 

Finally, we verified that an increase in cellular levels of p53 occurs by inhibiting the activity of p90RSK with BI-D1870 for a long period of time (4, 8, 10, 24, 48, 72 h). This is due to the prolonged BI-D1870-dependent dephosphorylation of serine 166 of MDM2 which, in addition to causing its destabilization, prevents it from degrading p53 (Figure 4H).

In conclusion, the results show that p90RSK is able to keep cellular levels of p53 low by increasing its turnover.

### 3.4. p90RSK Is Able to Regulate Proliferation and Apoptosis by Controlling p53 Levels

**Figure 5:**
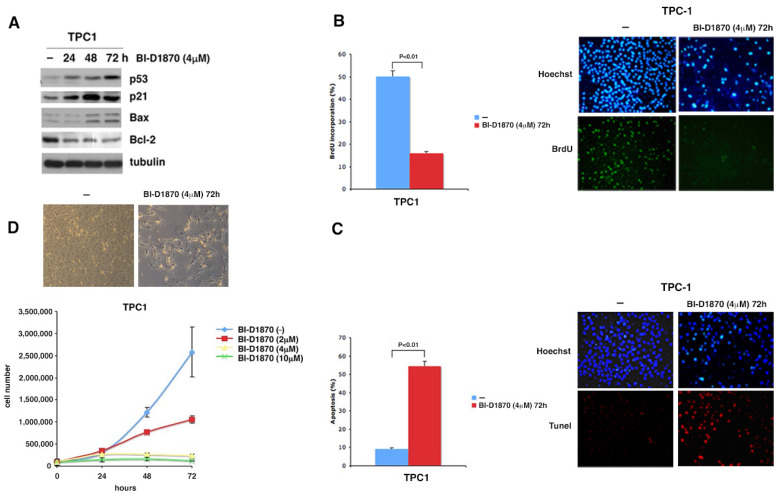
p90RSK is able to regulate proliferation and apoptosis by controlling p53 levels. (**A**) TPC1 cells were treated with 4 μM BI-D1870 in the indicated times (h). The immunoblot was tested with anti-p53, anti-p21, anti-Bax, anti-Bcl2 antibodies and normalized with the anti-tubulin antibody; (**B**) BrdU assay in TPC1 cells treated or not with 4 μM BI-D1870 for 72 h. Nuclei were stained with Hoechst (blue stain) and apoptotic cells were highlighted by anti-BrdU antibody (green stain). At least 500 cells per sample were counted. The percentage of BrdU positive cells was calculated as the ratio of BrdU-labeled nuclei to total nuclei. The experiment was conducted in triplicate. The histogram represents the mean value of positive BrdU cells among those counted in multiple fields (more than 100 cells counted in each field). The error was calculated with the standard deviation of the obtained values (left panel). The cells were photographed using a fluorescence microscope at 10× magnification. One representative field for sample was shown (right panel); (**C**) TUNEL assay in TPC1 cells treated or not with 4 μM BI-D1870 for 72 h. Total nuclei were stained with Hoechst (blue stain) and apoptotic cells were highlighted by the TUNEL test (red stain). The percentage of apoptotic cells was calculated as the ratio of red-labeled nuclei to total nuclei (blue). The histogram represents the mean value of apoptotic cells among those counted in multiple fields (more than 500 cells counted in total). The error was calculated with the standard deviation of the obtained values (left panel). The cells were photographed using a fluorescence microscope at 10× magnification. One representative field for sample was shown (right panel); (**D**) growth curve of TPC1 cells treated with different doses of BI-D1870. The experiment was conducted in triplicate. The results in the histogram are the average of the three independent determinations +/− standard deviation (lower panel). TPC1 cells, treated or not with 4µM BI-D1870 for 72 h, were photographed in the optical field at 10× magnification. A representative field was shown (upper panel).

p53 is known to regulate the transcription of several genes including the cell cycle inhibitor p21/WAF [27], the proapoptotic gene Bax [28] and the antiapoptotic gene Bcl-2 [39]. To verify if the increase in p53, due to the progressive S166 dephosphorylation of MDM2, leads to a change in the level of its targets, we treated TPC1 cells for 24, 48 and 72 h with BI-D1870 and performed a Western blot analysis. The gradual increase in p53 corresponds to a progressive increment in p21 and Bax and a progressive decrease in Bcl-2 (Figure 5A, supplementary Figure S5A). 

To define the effects on cells proliferation due to p53-dependent p21 increase, we treated TPC1 cells with BrdU after 72 h of treatment with 4 µM BI-D1870. A noticeable reduction in BrdU incorporation is evident in TPC1 cells treated with BI-D1870 (15.83%) compared to untreated cells (50.24%) (Figure 5B, supplementary Figure S5B) demonstrating that inhibition of p90RSK leads to a significant reduction in the replicative potential of TPC1 tumor cells, probably due to the increase in p21. 

Moreover, treatment with 4µM BI-D1870 for 72 h induces an increase in cell apoptosis, assessed by TUNEL assay in which cells treated with BI-D1870 showed an apoptotic rate of 54.4% compared to 9.2% of untreated cells (Figure 5C, supplementary Figure S5C). The increase in apoptotic rate perfectly agrees with the p53-dependent increment in Bax and reduction in Bcl-2 (Figure 5A, supplementary Figure S5A). Furthermore, a cytotoxic effect on TPC1 cells, due to 4 µM BI-D1870 treatment for 72 h, is also shown by the cellular microscopic image which highlights the cell suffering with reduced double refraction, structural cleavage and pycnotic nuclei (Figure 5D, supplementary Figure S5D, left panel). This alteration in cell morphology confirms the TUNEL results.

Finally, in a growth curve of TPC1 cells treated with different concentrations of BI-D1870 (2, 4, 10 µM), we show that inhibition of p90RSK leads to a strong reduction in cell growth (Figure 5D, supplementary Figure S5D, right panel). In fact, the number of cells after 72 h of treatment with 2 µM BI-D1870 is approximately half (1.297.500) compared to the number of untreated cells (2,557,500); this shows that BI-D1870, already at low doses, is able to induce a remarkable reduction of cell proliferation (Figure 5D, supplementary Figure S5D, lower panel). These data perfectly agree with BI-D1870-dependent inhibition of p90RSK, constitutively activated by the MAPK pathway in TPC1 cells.

**Figure 6:**
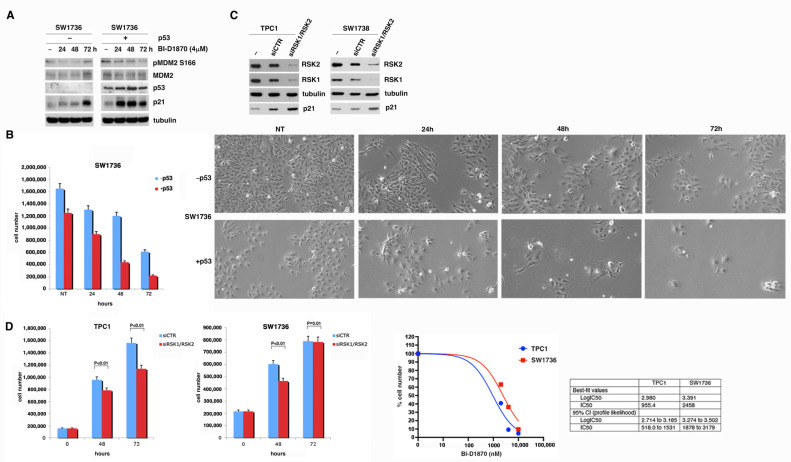
p53 contributes to control of p90RSK-dependent proliferation by regulating p21 expression. (**A**) SW1736 cells were transfected with p53 expression vector (+) or with the empty vector (−) and after 24 h treated or not (−) with 4 μM BI-D1870 for the indicated times (h). The efficiency of the transfection was verified with anti-p53 antibody. The effectiveness of the treatment was verified with anti-phospho-S166 MDM2 antibody. The immunoblot was then tested with anti-p21 and anti-MDM2 antibodies and normalization was performed by immunoblotting with anti-tubulin antibody. (**B**) SW1736 cells were transfected (+p53) or not (−p53) with p53 expression plasmid, treated or not (NT) with 4 μM BI-D1870 for the indicated times (hours) and counted. The experiment was conducted in triplicate. The histogram indicates the mean of the results for non-treated (NT), 24, 48, and 72 h and the calculated standard deviation was indicated (left panel). SW1736 (+/−p53) were then photographed in the optical field at 10× magnification. Representative fields were shown (right panel); (**C**) TPC1 (left panel) and SW1736 (right panel) cells were transfected with RNA interference against RSK1 and RSK2 (pool of specific RSK1/RSK2 siRNAs) or with control RNA interference (siCTR). The interference efficiency was verified with anti-RSK1 and anti-RSK2 antibodies. p21 total level was revealed with anti-p21 antibody. Normalization was performed by immunoblotting with anti-tubulin antibody. (**D**) Growth curve of TPC1 and SW1736 cells transfected with RNA interference against RSK1 and RSK2 (siRSK1/RSK2) or with control RNA interference (siCTR). The experiment was conducted in triplicate. The histogram indicates the mean of the results at 0, 48 h, and 72 h and the calculated standard deviation was indicated (left panels). Dose–response curve at 72 h of TPC1 and SW1736 cells treated or not with 2, 4, 10 μM BI-D1870 (right panel). BI-D1870 IC50 and 95% confidence interval (95% CI) calculated for TPC1 and SW1736 at 72 h of treatment were shown in the insert table.

To verify that the effect of BI-D1870 on p21 induction was strictly dependent on p53, we treated p53-null cells SW1736 [40] for different times (24, 48, 72 h) with 4 µM BI-D1870 and compared them with SW1736 transiently transfected with a p53wt expressing vector (SW1736-p53wt). The efficacy of the treatment was confirmed by the use of anti-pS166 MDM2 antibody. The result shows that in SW1736-p53wt, the increase in p21, dependent on the inhibition of p90RSK with BI-D1870, was stronger compared to that observed in the p53-null cells (Figure 6A). However, in SW1736 cells, a slight increase in p21 is visible anyway, most likely due to the contribution of the p73 protein, belonging to the p53 family, capable of transcriptionally regulating p21 in p53-null cells [41,42].

This result is perfectly in agreement with the effect of BI-D1870 (24, 48, 72 h) on the proliferation of SW1736 cells’ expression—or lack thereof—of exogenous p53wt (Figure 6B). Expression of exogenous p53wt results in a strong inhibition of proliferation relative to parental cells. However, the slight effect observed in SW1736 is most likely due to the contribution of p73 to the control of p53-null cell proliferation [43,44].

To better clarify the direct involvement of p90RSK in p21 regulation we performed a silencing of p90RSK with a siRNA pool (siRSK1/RSK2) in SW1736 and in TPC1 cells (Figure 6C). The results show a greater induction of p21 in TPC1 than that observed in SW1736. As mentioned above, a weak increase in p21 in SW1736 is in agreement with the contribution of p73 capable of transcriptionally regulating p21 in p53-null cells [41,42]. The observed increase in p21 agrees perfectly with a greater inhibition of proliferation, especially after 72 h of silencing of p90RSK with a siRNA pool (siRSK1/RSK2), in TPC1 compared to that observed in SW1736 cells (Figure 6D, left panel). Accordingly, the IC50 of BI-D1870 is higher in SW1736 compared to TPC1 (Figure 6D, right panel).

### 3.5. p90RSK Activation Is Associated to MDM2 Stabilization in TPC1 Cells and in Primary Thyroid Tumors

The data previously described are indicative of MDM2’s increased stabilization in thyroid tumor cell lines in which the p90RSK kinase is highly active. With an immunofluorescence assay, in which we detected the MDM2 protein using the specific antibody, we showed that the inhibition of p90RSK activity with BI-D1870 induces a reduction of MDM2 in the cell and, in particular, in the nucleus. In BI-D1870-treated cells, MDM2 decreased and is predominantly located in spots in the cytoplasm that could be presumably proteasomes. These data are in perfect agreement with those of other authors who demonstrate that the phosphorylation of S166 by AKT induces the translocation of MDM2 from the cytoplasm to the nucleus where it binds p53. In fact, S166 is present in the consensus motif RXRXXS/T p90RSK and is proximal to the sequences of the nuclear localization signal (NLS; amino acids 181–185) and nuclear export signal (NES; amino acids 190–200) of MDM2 [45].

Thus, we decided to analyze whether there was an association between p90RSK activation (anti-pRSK) and MDM2 expression levels in primary thyroid tumors.

For this purpose, we performed immunostaining for pRSK and MDM2 protein in 13 thyroid tumors (9 PTC, 2 FTC and 2 ATC). We found nine tumors positive for pRSK; in MDM2 was clearly detectable eight of them (88.9% match), one was negative; four tumors were negative for pRSK and were also negative for MDM2 staining except for one of them (75% match). Using Fisher’s exact test, we established that there is a significant association between p90RSK phosphorylation and MDM2 detection in the analyzed tumors: the percentage of tumors positive for MDM2 was significantly greater in the high pRSK level group and MDM2-negative group was significantly greater in tumors negative for pRSK expression (*p* < 0.05). Thyroid carcinomas were classified as positive for MDM2 expression and for RSK phosphorylation when they exhibited diffuse nuclear/cytosolic staining in several cells (cut-off fixed at >5% of tumor cells compared to normal cell). 

**Figure 7:**
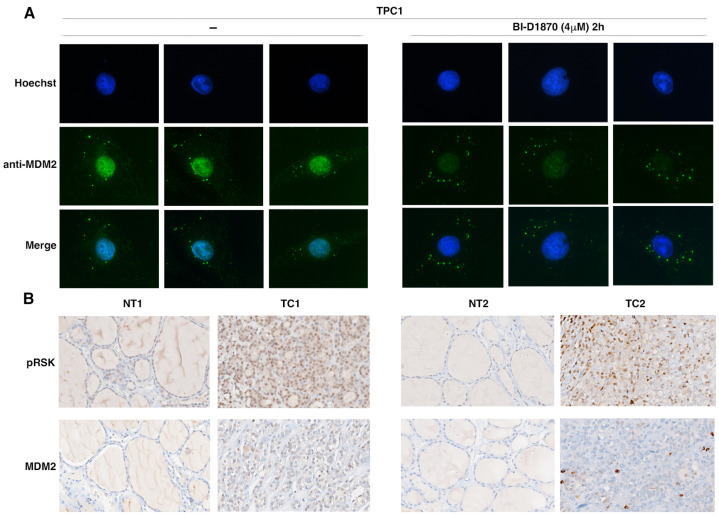
p90RSK activation is associated with MDM2 stabilization in TPC1 cells and in primary thyroid tumors. (**A**) Staining of MDM2 by immunofluorescence assay in TPC1 cells treated or untreated with 10 μM BI-D1870 for 2 h. Untreated TPC1 cells show strong nuclear staining for MDM2; BI-D1870-treated TPC1 cells show reduced and predominantly cytosolic staining for MDM2. Hoechst staining was used to visualize cell nuclei. The experiment was performed in triplicate, and at least 90 representative cells were observed. Representative fields of treated and untreated cells are shown. Magnification 100×; (**B**) immunohistochemical staining for MDM2 and pRSK of formalin-fixed, paraffin-embedded thyroid tissue samples. Tissue samples from normal thyroid (NT) or thyroid carcinoma (TC) were incubated with a mouse monoclonal anti-MDM2 and anti-pRSK antibodies. TCs show diffused cytoplasmic positivity with nuclear accumulation for pRSK, whereas NTs show only rare elements with weakly nuclear positivity. TCs show diffused cytoplasmic positivity with nuclear accumulation for MDM2, whereas corresponding NTs are negative. Representative pictures of normal and pathologically positive samples are shown. Magnification 40×.

Figure 7 shows representative immunostaining.

## 4. Discussion

In this manuscript we demonstrated that p90RSK binds and phosphorylates MDM2 on S166 in vitro and in vivo, resulting in the release of phosphorylated MDM2 from the p90RSK/MDM2 complex. Through this mechanism, p90RSK is able to maintain low cellular levels of p53 by increasing its turnover, dependent on MDM2 serine 166 phosphorylation. P53, by transcriptionally regulating the cell cycle inhibitor p21/WAF, the proapoptotic gene Bax, and the antiapoptotic gene BCL2, regulates the proliferation and apoptosis of TPC1 cells for which p90RSK proves to play a key role in the regulation of these two biological processes in our tumor model. Finally, even if on a small number of samples, we demonstrated the existence of a correlation between p90RSK activation and MDM2 phosphorylation in primary thyroid tumors. Since targeted therapy, which works by blocking a specific molecular target to inhibit tumor progression and metastasis, is a type of strategy widely used for the treatment of many types of cancer [46], the identification of the right molecular targets, such as p90RSK, on which to act pharmacologically is fundamental to developing successful targeted therapies.

In many human cancers, including thyroid, breast, lung, colorectal cancers, and melanoma, the MAPK pathway is constitutively activated through mutations of its effectors: tyrosine kinases receptor (RTKs), their direct targets such as RAS, or downstream kinases belonging to the MAPK pathway [47,48,49,50,51,52,53,54,55,56]. Usually, mutations in the MAPK pathway are mutually exclusive and there are not mutations in different genes of the same tumor [47,57,58]. The dysregulation of kinase activity and hyperactivation of the MAPK pathway induct progression of tumorigenesis, influencing cell proliferation and invasion [59].

For this reason, the use of MAPK pathway inhibitors represents an important anticancer strategy. BRAF inhibitors (BRAFi), such as dabrafenib and vemurafenib, have shown excellent response rates and, in addition, MEK1/2 inhibitors, such as trametinib, binimetinib, and cobimetinib, have been approved in melanoma and non-small cell lung cancer with mutated BRAF [57,58]. However, the duration of response to these inhibitors is limited because, within 6–8 months, a resistance mechanism is developed due to genetic mutations, including MEK1/2 mutations, BRAF amplification, BRAF alternative splicing, and NRAS mutations and/or to epigenetic modifications, resulting in reactivation of MAPK or alternative pathways such as PI3K/Akt [60,61,62,63,64,65]. The BRAF V600E mutation is the most common mutation in thyroid cancers, and it is often associated with tumor aggressiveness and a poor prognosis. Thyroid tumors positive of BRAFV600E are completely resistant or show a poor response to radioactive iodine therapy because radioiodine uptake is inhibited in them. Therefore, the use of molecule-targeted BRAF in BRAF-mutated, advanced or metastatic, radioactive iodine (RAI)-resistant thyroid cancer has become a priority. However, many thyroid cancers show primary or acquired resistance to all BRAF inhibitors available to date [66].

Initially, it was thought that the combination of BRAF and MEK inhibitors could be useful in evading resistance mechanisms, improving patients’ progression-free and overall survival; however, the use of this therapeutic combination proved to develop the same resistance mechanisms that are generated following the use of a single drug, after about one year of treatment [62,63]. Since in some tumors, such as melanoma, BRAF and MEK inhibitors are frequently used and resistance mechanisms develop several months after their use, the identification of inhibitors that allow the possibility to block alternative targets of the tumor, regulating cell cycle and apoptosis, may represent an important goal and can be considered for the design of new combinatorial therapies based to the inhibition of multiple targets at the same time [67].

The p90RSK family represents a downstream effector of the MAPK pathway and activation of the p90RSK signal is involved in many types of tumors, regulating different oncogenic processes through a series of substrates involved in transcription, translation, cell cycle regulation, and cell survival [7,8]. Hence, these kinases are a very promising target for possible inhibitors to regulate tumor cell invasion and metastasis and thus could be added to current anticancer therapies [68]. 

However, despite the use of p90RSK inhibitors as part of a therapeutic cocktail, it should guarantee fewer side effects, as p90RSK controls fewer processes than its upstream activators (B-RAF, MEK, ERK) [69]; currently, available p90RSK inhibitors act simultaneously on multiple isoforms (pan-RSK) and there are still no specific for each isoform of drugs that have pharmacokinetic properties to be used in vivo. This represents a major limitation for their efficacy as anticancer agents due to the toxic effects induced by their use [15,70].

Furthermore, it is important to consider that although RSKs have a large homology in the protein sequence, each isoform performs a different, sometimes opposite, function in various types of cancer and therefore the use of pan-RSK is not always useful.

These considerations suggest that selective inhibition of specific p90RSK isoforms represents a more promising way of targeting RSKs in cancer. 

Therefore, although we focused on the regulation of p53 by MDM2 in this manuscript, a mechanism already known and described for some time, we introduced a new fundamental node represented by the kinase p90RSK, which makes this mechanism the basis of a possible new therapeutic target for many types of cancer. The S166 is a very important site for MDM2. Indeed, various kinases phosphorylate this serine, among them we already mentioned PKB/AKT in the introduction section [38]. Malmlöf introduces a fundamental concept: they show that in hepatocarcinoma the phosphorylation of MDM2 on serine 166 is dependent on the activation of the MAPK pathway; in fact it, is inhibited by the action of PD98059 or U0126 (MEK inhibitors), but not by LY-294002 or Wortmannin (PI3K inhibitors), while the reverse occurs in lung cancer, as whether this phosphorylation appears depends on the PI3K pathway [71].

This suggests that the pathway influencing S166 phosphorylation of MDM2 and, consequently, that p53 expression is tumor type-dependent. In any tumor, this phosphorylation may depend on a pathway that is mainly dysregulated and influences tumorigenesis. In thyroid cancer, MAPK dysregulation plays an important role in tumor promotion and progression. For this reason, it is important to study the effect of this specific pathway on S166 phosphorylation of MDM2 and how it affects the amount of p53.

## 5. Conclusions

**Figure 8:**
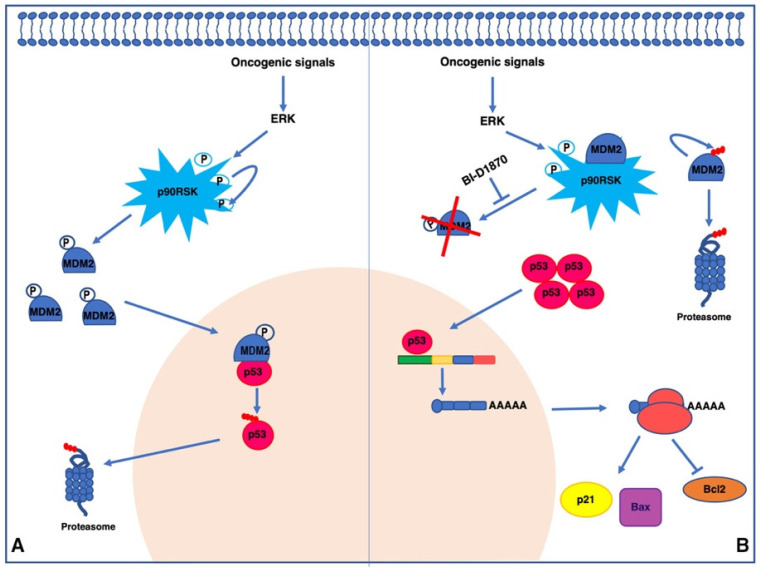
Final model of p53 degradation dependent on MDM2 phosphorylation by p90RSK. (**A**) In tumor cells, oncogenic signals lead to the activation of p90RSK via ERK. Activated p90RSK is capable of phosphorylating itself and various substrates including the ubiquitin ligase MDM2 on serine 166. This phosphorylation protects MDM2 from degradation, stabilizing it. MDM2, in turn, induces ubiquitination and proteasome-mediated degradation of p53; (**B**) treatment with the p90RSK inhibitor BI-D1870 destabilizes MDM2, which is released from the MDM2/p90RSK complex and leads to self-degradation through the proteasome. This reduces the levels of MDM2 and leads to a simultaneous increase in p53 that, in turn, increases the transcription of p21 and Bax and reduces the transcription of Bcl-2.

In this manuscript we demonstrated a novel p90RSK-mediated p53 regulation mechanism (Figure 8). p90RSK binds and phosphorylates MDM2 on serine 166, increasing the proteasome-dependent degradation of p53. Since p53 transcriptionally regulates the expression of the cell cycle inhibitor p21, the pro-apoptotic protein Bax, and the anti-apoptotic protein Bcl-2, p90RSK activity influences cell proliferation and apoptosis. Thus, p90RSK inhibition induces p53 increase, downregulating cell proliferation and increasing cell apoptosis, making p90RSK a very attractive molecular target for the development of new anticancer drugs.

## Data Availability

Not applicable.

## Supplementary Materials

The following supporting information can be downloaded at: https://www.mdpi.com/article/10.3390/cancers15010121/s1, Figure S1: p90RSK binds and phosphorylates MDM2 on S166 in vivo; Figure S2: Western blot with quantization for Figure S1; Figure S3: Western blot with quantization for Figure 3; Figure S4: Western blot with quantization for Figure 4; Figure S5: Western blot with quantization for Figure 5; Figure S6: In HeLa cells p53 regulation by p90RSK appears to be influenced by MDM2 phosphorylation on S166; Figure S7: Role of PI3K/AKT pathway in degradation of p53 via MDM2 phosphorylation in TPC1 cells.
