# Supplementary figures and images for: p90RSK Regulates p53 Pathway by MDM2 Phosphorylation in Thyroid Tumors
Source: Cancers (Basel). 2022 Dec 25;15(1):121. doi: 10.3390/cancers15010121 (PMC9817759; doi:10.3390/cancers15010121)

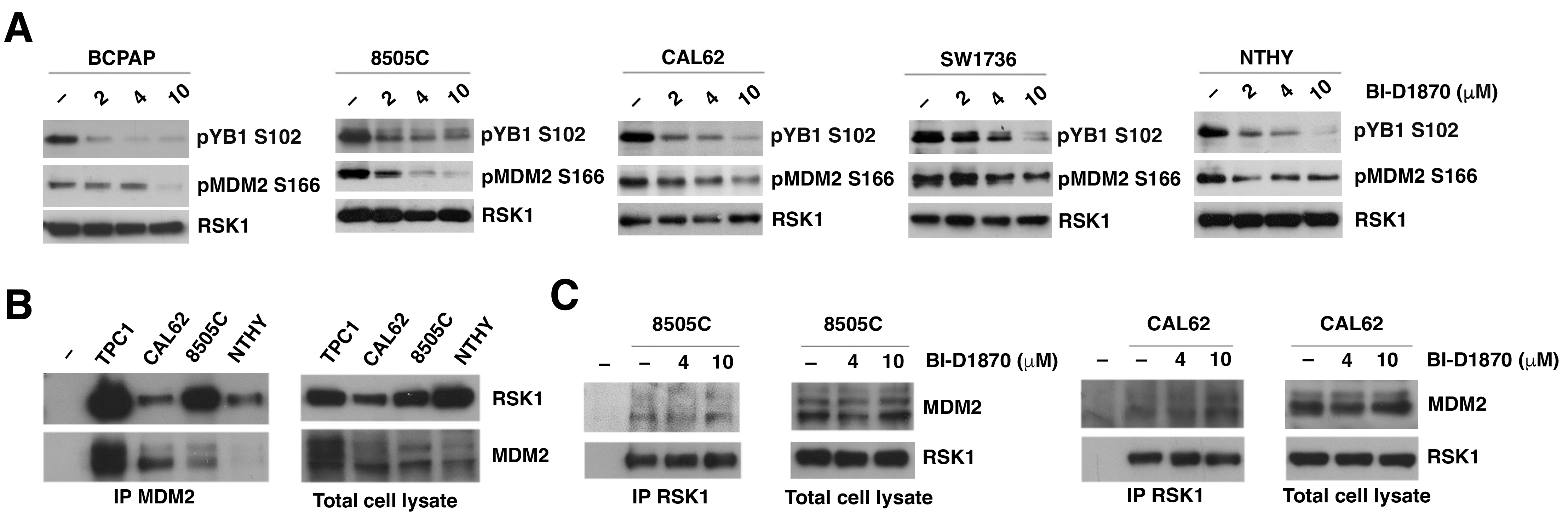

Supplement: Supplementary file 1 [file cancers-15-00121-s001.zip › cancers-1999963-SI/Fig S1 pr.jpg]

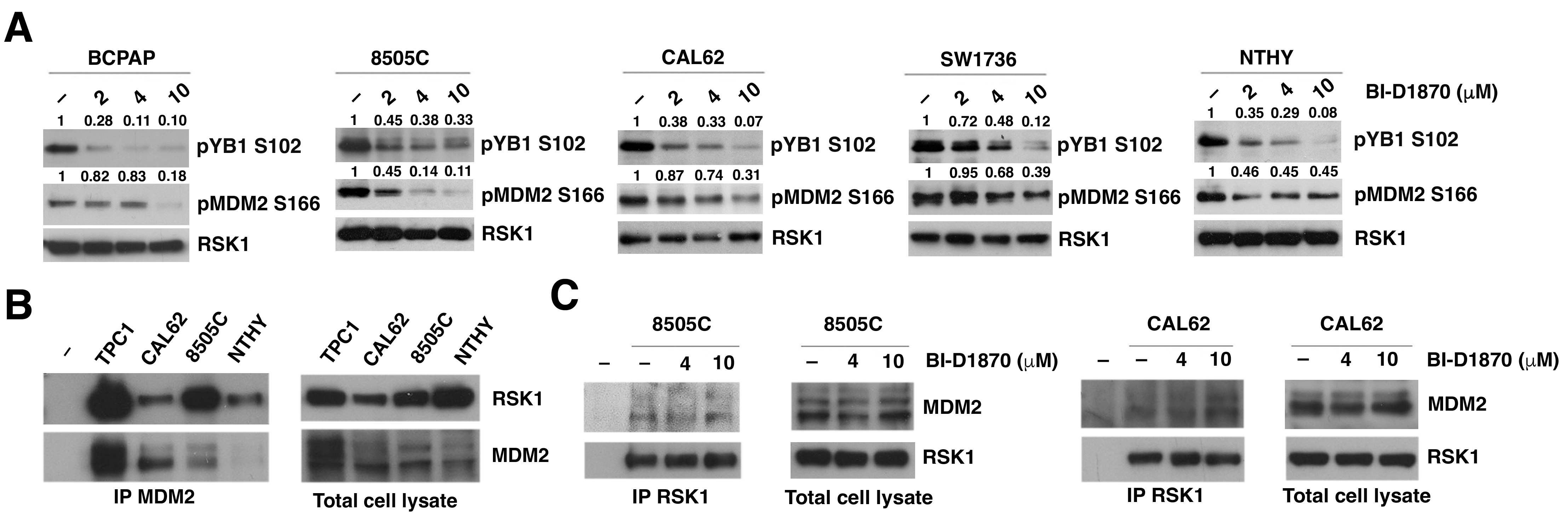

Supplement: Supplementary file 1 [file cancers-15-00121-s001.zip › cancers-1999963-SI/Fig S2 pr.jpg]

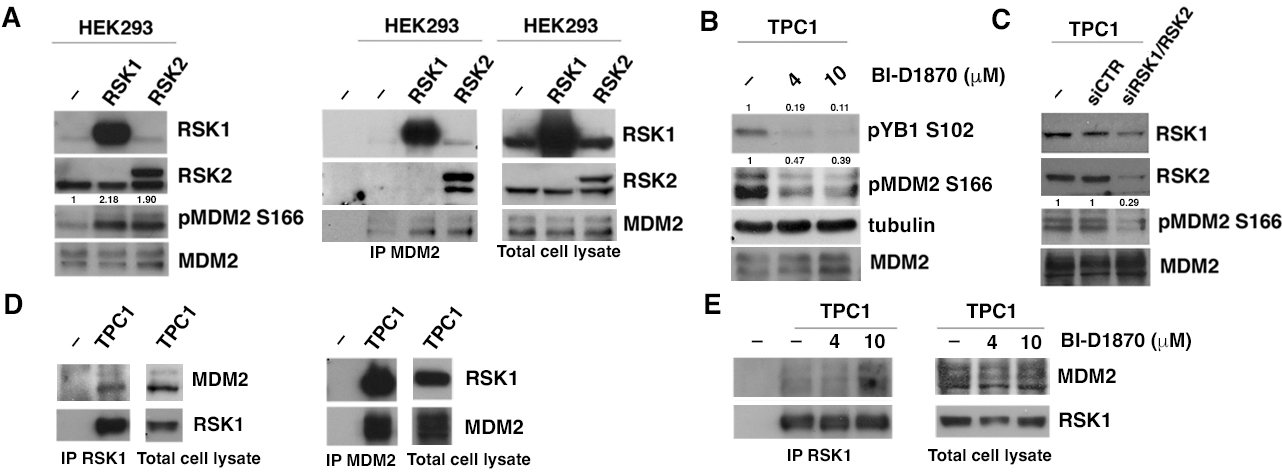

Supplement: Supplementary file 1 [file cancers-15-00121-s001.zip › cancers-1999963-SI/Fig S3 pr.jpg]

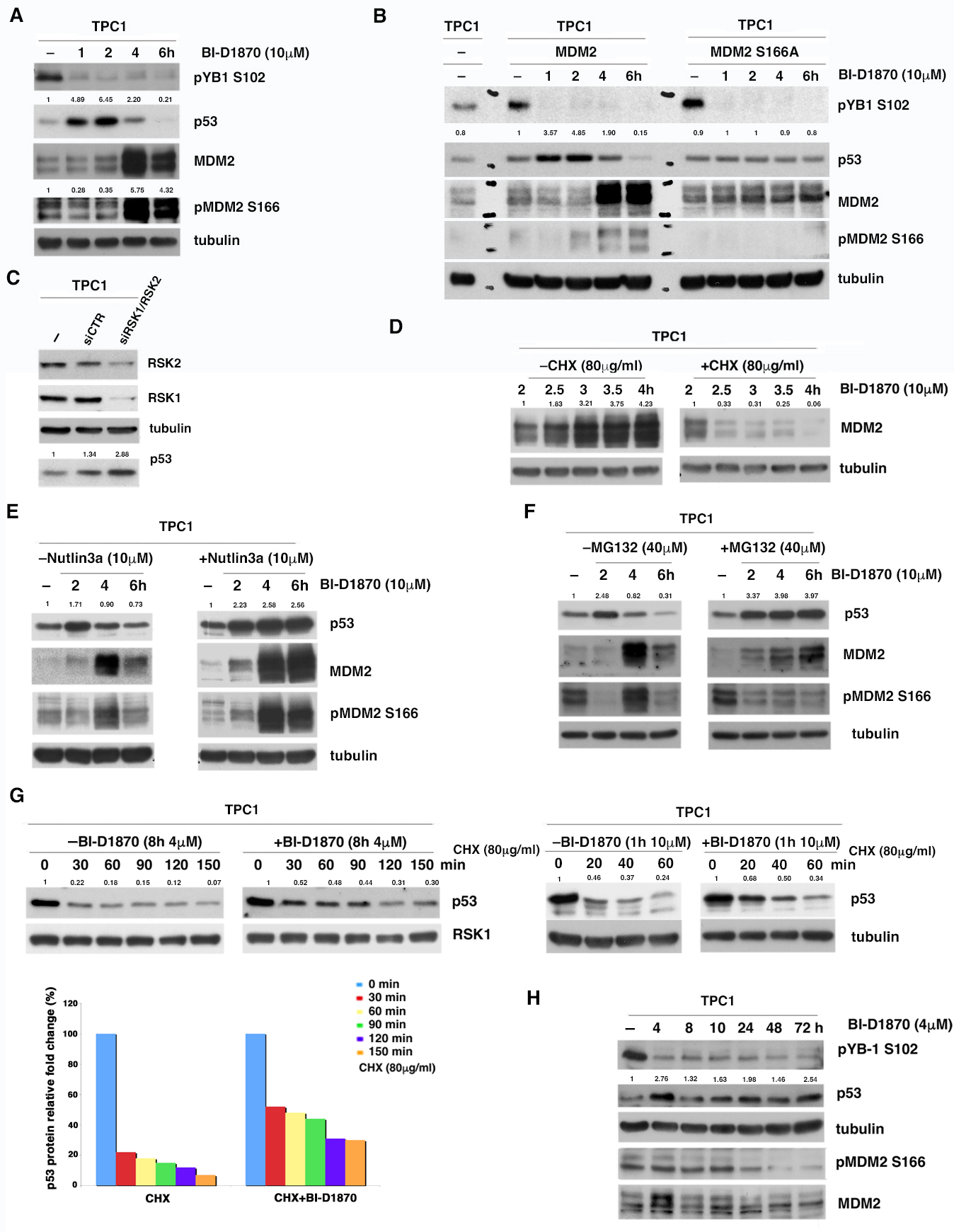

Supplement: Supplementary file 1 [file cancers-15-00121-s001.zip › cancers-1999963-SI/Fig S4 pr.jpg]

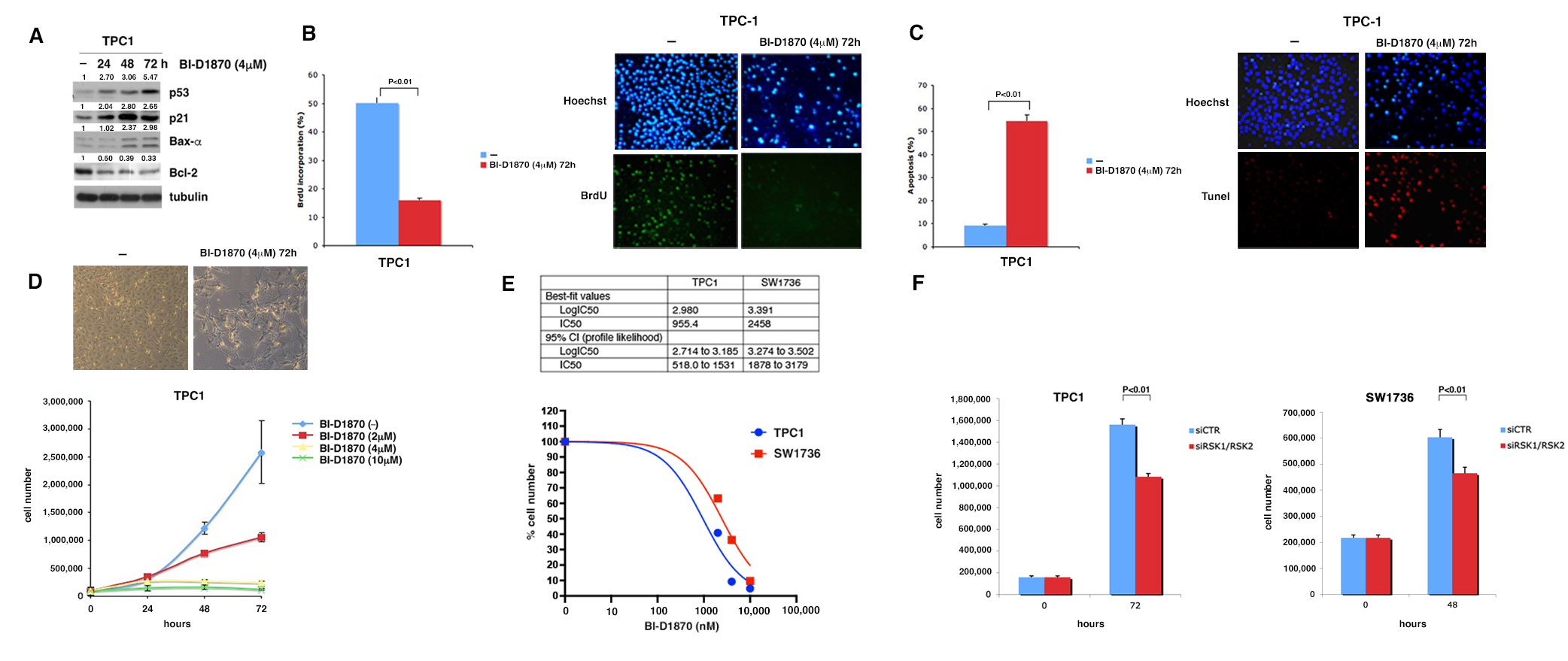

Supplement: Supplementary file 1 [file cancers-15-00121-s001.zip › cancers-1999963-SI/Fig S5 pr.jpg]

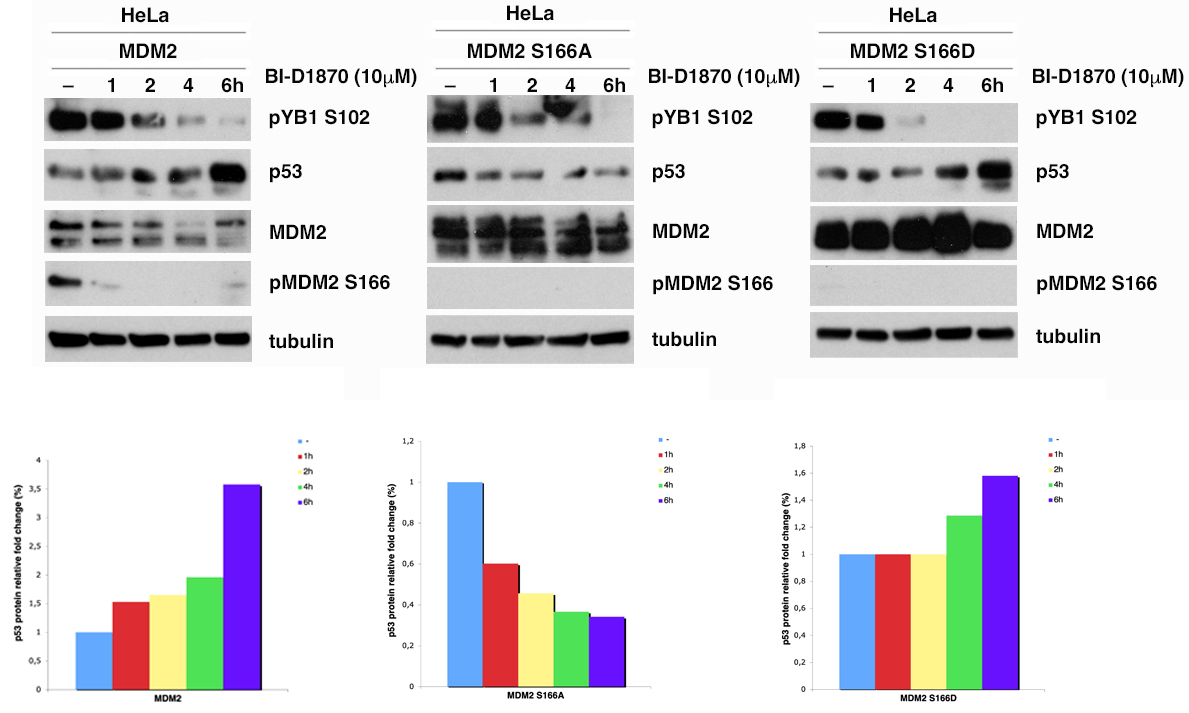

Supplement: Supplementary file 1 [file cancers-15-00121-s001.zip › cancers-1999963-SI/Fig S6 pr.jpg]

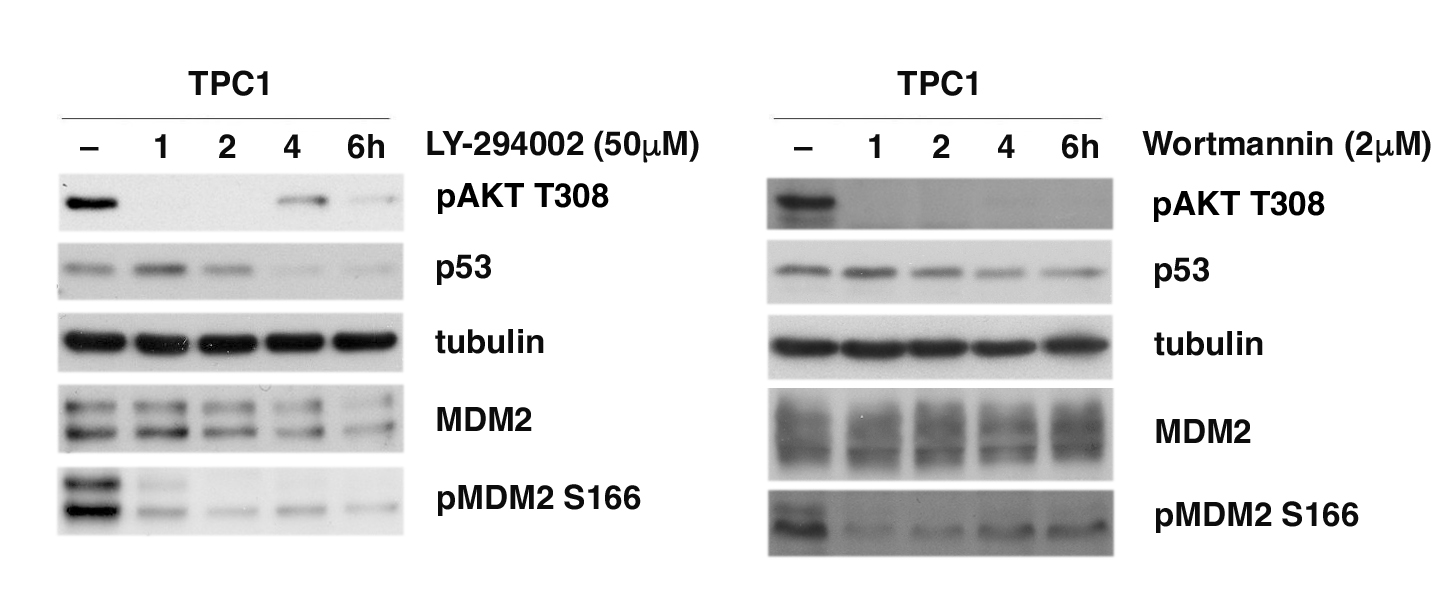

Supplement: Supplementary file 1 [file cancers-15-00121-s001.zip › cancers-1999963-SI/Fig S7 pr.jpg]
